# Formoterol Exerts Anti-Cancer Effects Modulating Oxidative Stress and Epithelial-Mesenchymal Transition Processes in Cigarette Smoke Extract Exposed Lung Adenocarcinoma Cells

**DOI:** 10.3390/ijms242216088

**Published:** 2023-11-08

**Authors:** Maria Ferraro, Serena Di Vincenzo, Valentina Lazzara, Paola Pinto, Bernardo Patella, Rosalinda Inguanta, Andreina Bruno, Elisabetta Pace

**Affiliations:** 1 Institute of Translational Pharmacology (IFT), National Research Council (CNR), 90146 Palermo, Italy; serena.divincenzo@ift.cnr.it (S.D.V.); andreina.bruno@ift.cnr.it (A.B.); elisabetta.pace@ift.cnr.it (E.P.); 2 Dipartimento di Scienze Economiche, Aziendali e Statistiche, Università degli Studi di Palermo, 90100 Palermo, Italy; valentina.lazzara@you.unipa.it; 3 Dipartimento di Sanità Pubblica, Medicina Sperimentale e Forense, Università di Pavia, 27100 Pavia, Italy; paola.pinto01@universitadipavia.it; 4 Laboratorio di Chimica Fisica Applicata, Dipartimento di Ingegneria, Università di Palermo, 90128 Palermo, Italy; bernardo.patella@unipa.it (B.P.); rosalinda.inguanta@unipa.it (R.I.)

**Keywords:** lung cancer, oxidative stress, cigarette smoke, inflammation, EMT, formoterol

## Abstract

Lung cancer frequently affects patients with Chronic Obstructive Pulmonary Disease (COPD). Cigarette smoke (CS) fosters cancer progression by increasing oxidative stress and by modulating epithelial-mesenchymal transition (EMT) processes in cancer cells. Formoterol (FO), a long-acting β2-agonist widely used for the treatment of COPD, exerts antioxidant activities. This study explored in a lung adenocarcinoma cell line (A549) whether FO counteracted the effects of cigarette smoke extract (CSE) relative to oxidative stress, inflammation, EMT processes, and cell migration and proliferation. A549 was stimulated with CSE and FO, ROS were evaluated by flow-cytometry and by nanostructured electrochemical sensor, EMT markers were evaluated by flow-cytometry and Real-Time PCR, IL-8 was evaluated by ELISA, cell migration was assessed by scratch and phalloidin test, and cell proliferation was assessed by clonogenic assay. CSE significantly increased the production of ROS, IL-8 release, cell migration and proliferation, and SNAIL1 expression but significantly decreased E-cadherin expression. FO reverted all these phenomena in CSE-stimulated A549 cells. The present study provides intriguing evidence that FO may exert anti-cancer effects by reverting oxidative stress, inflammation, and EMT markers induced by CS. These findings must be validated in future clinical studies to support FO as a valuable add-on treatment for lung cancer management.

## 1. Introduction

Lung cancer represents the leading cause of morbidity and mortality worldwide [1]. The frequent recurrence of COPD in lung cancer patients and the finding that COPD patients showed a 6-fold increase in the risk of developing lung cancer compared to smokers [2] demonstrate a possible relationship between COPD and lung cancer [3]. Histologically, lung cancer is classified as small-cell lung cancer (SCLC) and non-SCLC (NSCLC). NSCLC is further classified into adenocarcinoma and squamous cell carcinoma (SCC). Adenocarcinoma is prevalent in patients with COPD and GOLD (Global Initiative for Chronic Obstructive Lung Disease) Stage I, while squamous cell carcinoma is the most frequent in GOLD, Stages II and III [4]. Cigarette smoke (CS) is the main risk factor for COPD and lung cancer [5] and is an aerosol composed of a gas phase and a particle phase. It is enriched by different chemical components with toxic, carcinogenic, and oxidant activity. CS causes inflammation and oxidative stress with damage to cell membrane lipids through various mechanisms, such as DNA damage and lipid peroxidation [6]. It can also alter the physical barrier, resulting in increased permeability of respiratory epithelial cells that leads to epithelial-mesenchymal transition (EMT). During the EMT process, epithelial cells lose their polarity and transform themselves into mobile mesenchymal cells [7].

EMT has a central role not only in embryonic development but also in wound healing, tissue regeneration, cancer metastasis, and tissue fibrosis [8]. Recent studies have highlighted the association between smoking-related COPD and EMT, and this process is associated with a higher prevalence of lung cancer, suggesting the importance of EMT not only in the development of COPD [9] but also in promoting lung cancer progression and metastatic expansion [10]. The main morphological characteristics of the EMT process are (a) the disruption of epithelial cell junctions, (b) the loss of polar complexes, and (c) the rearrangement of the structures that make up the cytoskeleton. The hallmarks of this process are the downregulation of epithelial junction proteins (e.g., E-cadherin and Occludins) and activation of EMT transcriptional activators (e.g., SNAIL1, SLUG, and TWIST) as well as the up-regulation of mesenchymal markers (Vimentin, Fibronectin, and N-cadherin) [11]. E-cadherin has a central role in the EMT process because it is involved in interepithelial junctions, and its repressed expression is linked to the activity of Snail, a transcription factor with a crucial role in multiple signaling pathways leading to EMT. The expression of SNAIL is closely associated with cancer metastasis [10]. 

It is known that CS could induce EMT in the alveolar type II adenocarcinoma cell line A549 [12]. A recent study showed that CS interferes with the normal EMT process [13], preventing the restoration of tissue homeostasis following damage to the airways. Several studies have demonstrated a marked reduced expression of E-cadherin in smokers and COPD [14]. COPD is characterized by airway inflammation, and CS induces the release of many inflammatory mediators. In particular, IL-8 is closely related to oxidative stress pathways [15]. High levels of IL-8 contribute to the tumorigenesis process in airway cells exposed to cigarette smoke extract (CSE) [16] and promote cancer cell invasion and metastasis [17]. Furthermore, a close correlation exists between the activation of the IL-8/IL-8R axis and the EMT process [18]. Long-acting β2-agonists (LABAs) are indicated for regular treatment of symptomatic patients with moderate-to-severe COPD. They act by relaxing airway smooth muscle by stimulating β2-adrenergic receptors, and this is associated with an increase of the intracellular messenger cyclic adenosine monophosphate (cAMP) [19]. Formoterol (FO) is a LABA that counteracts inflammation processes induced by the exposure of bronchial epithelial cells to cigarette smoke [20] and reduces mitochondrial oxidative stress induced by CSE in the bronchial epithelial cell line, enhancing in this way the anti-inflammatory effect of corticosteroid [21]. A high level of cAMP counteracts the EMT process [22]. The combination of LABA, formoterol, and the muscarinic receptor antagonist (LAMA), vilanterol, inhibits the ECM process through cAMP [23].

To our knowledge, no evidence has been reported via clinical studies on the therapeutical role of FO in lung cancer; based on this, the aim of this study was to assess for the first time whether FO can revert some processes related to oxidative stress, EMT, and cancer progression induced by exposure to CS. We evaluated in an adenocarcinoma cell line model (A549) exposed to CSE, oxidative stress (intracellular and extracellular ROS), inflammation (IL-8), EMT processes (E-cadherin, SNAIL1), and cell migration and proliferation.

## 2. Results

### 2.1. Effects of CSE and FO on Intracellular Oxidative Stress and Intracellular ATP in A549 

**Figure 1:**
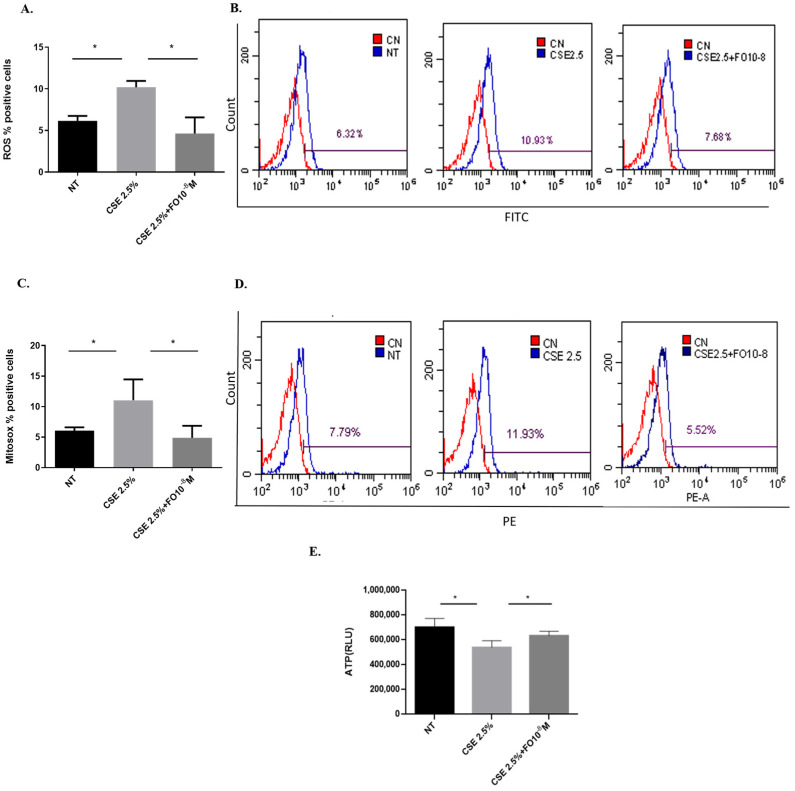
ROS, mitochondrial superoxide, and intracellular ATP production in A549 cells. A549 cells were exposed to CSE 2.5% with or without FO 10^−8^ M for 24 h. FO was added 30 min before CSE cell stimulation. ROS, mitochondrial superoxide, and intracellular ATP production were assessed by flow cytometry and the luminescence method. See the Section 4 for further details. ROS (**A**) and mitochondrial superoxide (**C**) levels in A549 cells are shown as % positive cells, and intracellular ATP production (**E**) is shown as Relative Luminescence Units (RLU) and expressed as mean ± SD (*n* = 5). Representative histograms of relative ROS (**B**) and mitochondrial superoxide (**D**) analysis are shown. * *p* < 0.05 ANOVA with Bonferroni correction.

Increased oxidative stress can be considered a useful feed for cancer cell growth [24]. Since CSE is able to increase oxidative stress in lung cancer cells [25], the effects of CSE and FO on intracellular ROS production and superoxide mitochondrial production were tested. As shown in Figure 1A, CSE was able to significantly increase intracellular ROS, and pre-incubation with FO was able to significantly decrease this CSE-mediated effect. In addition, FO was able to significantly revert the effects of CSE on the increased production of the mitochondrial superoxide (Figure 1C). We also evaluated intracellular oxidative stress, ROS production, and superoxide mitochondrial production in another adenocarcinoma cell line, COLO699 N, and we obtained results similar to those observed in the A549 cell line, Figure S3. Mitochondrial damage induced by CS is associated with a reduction of intracellular ATP levels [26]. CSE was able to significantly reduce intracellular ATP, and FO was able to significantly revert the effects of CSE, reducing intracellular ATP production.

### 2.2. Effects of CSE and FO on Extracellular Oxidative Stress in A549 

**Figure 2:**
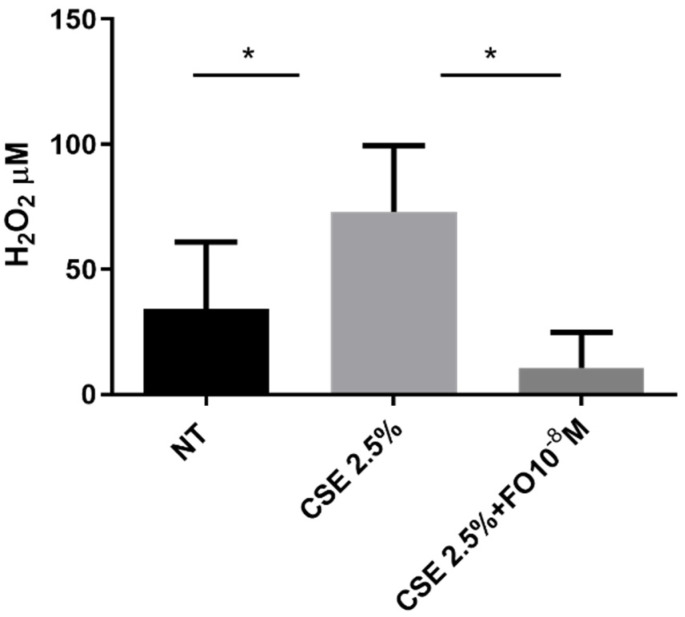
Extracellular oxidative stress in A549 cells. A549 cells were exposed to CSE 2.5% with or without FO 10^−8^ M for 24 h. FO was added 30 min before CSE cell stimulation. Extracellular oxidative stress was evaluated by detecting hydrogen peroxide in an electrochemical sensor. See the Section 4 for further details. Levels of H_2_O_2_ are reported in μM and expressed as mean ± SD (*n* = 7). * *p* < 0.05 ANOVA with Bonferroni correction.

The extracellular oxidative stress was evaluated using an innovative electrochemical sensor that, as demonstrated [27], was able to detect hydrogen peroxide. The sensor was used to quantify hydrogen peroxide in cell supernatant collected from CSE- and/or FO-stimulated A549 cells. Data shown in Figure 2 demonstrated that CSE was able to significantly increase extracellular oxidative stress and that FO was able to significantly counteract this effect.

### 2.3. Effects of CSE and FO on IL-8 Release by A549 

**Figure 3:**
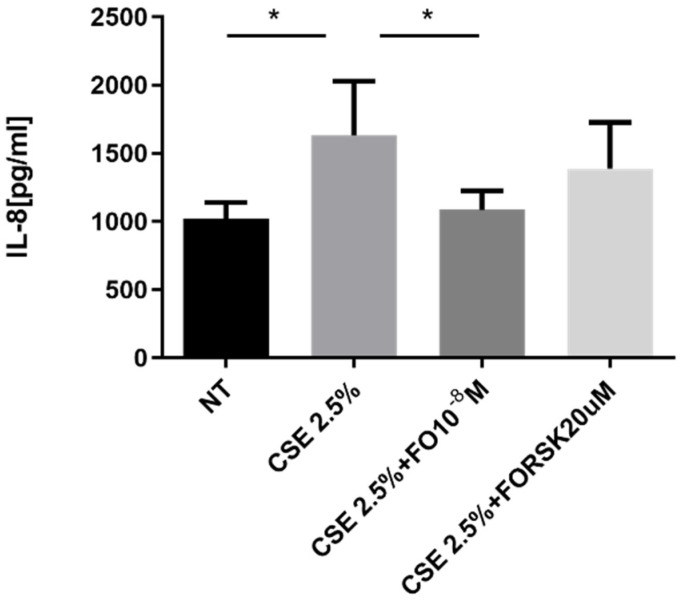
IL-8 release in A549 cells. A549 cells were exposed to CSE 2.5% with or without FO 10^−8^ M or FORSK 20 μM for 24 h. FO and FORSK were added 30 min before CSE cell stimulation. See the Section 4 for further details. IL-8 release was evaluated by ELISA. Levels of IL-8 are reported in pg/mL and expressed as mean ± SD (*n* = 5). * *p* < 0.05 ANOVA with Bonferroni correction.

The pro-inflammatory cytokine IL-8 represents one of the key promoters of tumor progression [28], and its production is increased by elevated oxidative stress [16]. A549 cells stimulated by CSE released significantly increased levels of IL-8, and pre-incubation with FO significantly restored the basal levels of IL-8 that were increased by CSE (Figure 3). After the binding of FO to beta-adrenergic receptors, there was activation of adenylate cyclase and an increase in cAMP. We introduced forskolin (FORSK) as an internal control because it causes an increase in cAMP levels. A549 cultured with CSE and forskolin tended to release less IL-8 than CSE-stimulated cells (Figure 3).

### 2.4. Effects of CSE and FO on the E-Cadherin Protein and Gene Expression in A549

**Figure 4:**
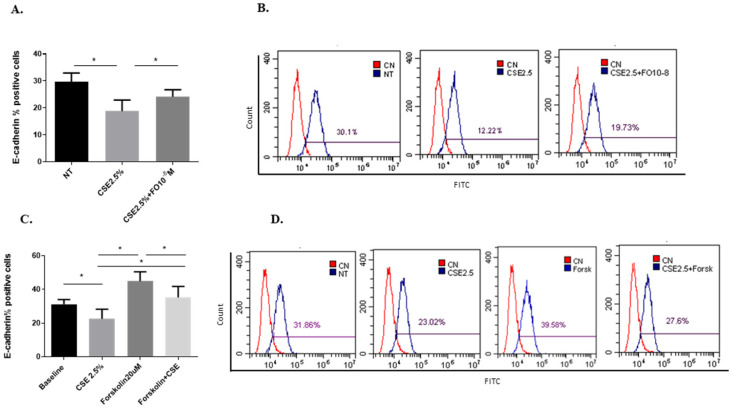
E-cadherin protein expression in A549 cells. A549 cells were exposed to CSE 2.5% with or without FO 10^−8^ M (**A**) or FORSK 20 μM (**C**) for 48 h. FO or FORSK were added 30 min before CSE cell stimulation. E-cadherin protein expression was evaluated by flow cytometry. See the Section 4 for further details. E-cadherin protein expression in A549 cells is shown as % positive cells and expressed as mean ± SD (*n* = 5). Representative histograms relative to E-cadherin protein expression (**B**–**D**) are shown. * *p* < 0.05 ANOVA with Bonferroni correction.

**Figure 5:**
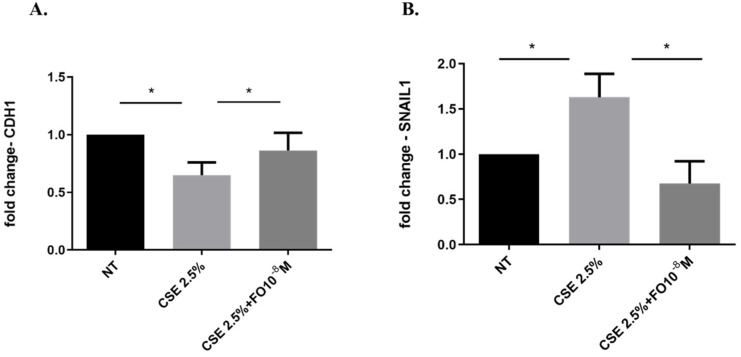
CDH1 and SNAIL1 gene expression in A549 cells. A549 cells were exposed to CSE 2.5% with or without FO 10^−8^ M for 24 h. FO was added 30 min before CSE cell stimulation. See the Section 4 for further details. CDH1 (**A**) and SNAIL1 (**B**) gene expression were evaluated by RT-PCR. Results are shown as relative units, expressed as mean ± SD (*n* = 5), and normalized to the untreated sample (NT). * *p* < 0.05 ANOVA with Bonferroni correction.

Epithelial-mesenchymal transition (EMT) processes play a determining role in cancer progression [29]. One of the main and early events in the EMT is represented by E-cadherin down-regulation [30]. CSE reduced both E-cadherin protein (Figure 4A,B) and gene (Figure 5A) expression. Pre-incubation with FO significantly increased E-cadherin protein (Figure 4A) and gene (Figure 5A) expression. Forskolin mimicked the FO effects in CSE-exposed cells (Figure 4C).

### 2.5. Effects of CSE and FO on SNAIL1 Gene Expression in A549 

SNAIL1 is a transcription factor that, once activated, reduces E-cadherin expression [31]. CSE significantly increased SNAIL1 gene expression (Figure 5B). Pre-incubation with FO significantly decreased SNAIL1 gene expression (Figure 5B).

### 2.6. Effects of CSE and FO on Cell Migration and Proliferation in A549

**Figure 6:**
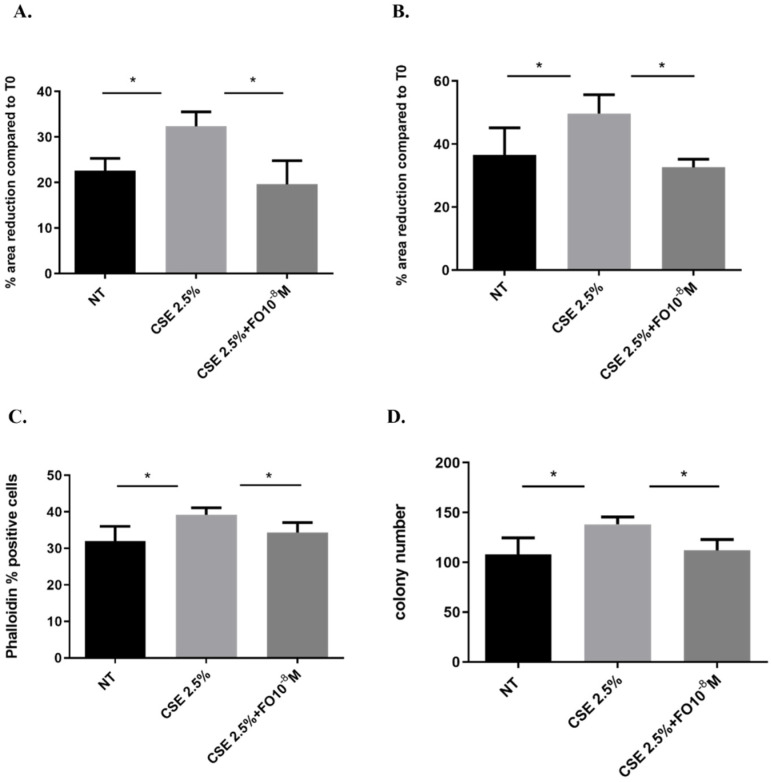
Cell migration and proliferation in A549 cells. A549 cells were exposed to CSE 2.5% with or without FO 10^−8^ M for 24 h. FO was added 30 min before CSE cell stimulation. For the scratch assay (**A**,**B**), the results are expressed as a percentage of area reduction at 24 h (**A**) and 48 h (**B**) compared to time point 0 h (*n* = 6). For actin reorganization, the expression of F-actin was evaluated using a FITC-Phalloidin probe via flow cytometry (**C**) (*n* = 6). For the clonogenic assay, the results are expressed as colony number (**D**) (*n* = 4). See the Section 4 for further details. * *p* < 0.05 ANOVA with Bonferroni correction.

EMT is a process that induces a change from a non-motile epithelial to a motile mesenchymal state [32] and promotes cells to be more invasive with a higher colony-forming capacity [25]. We assessed whether CSE and FO altered cell migration by evaluating a scratch assay and measuring F-actin using the FITC-phalloidin probe and proliferation by clonogenic assay. CSE increased cell migration in A549 at 24 and 48 h, and pre-incubation with FO was able to restore basal cell migration at both tested time points (Figure 6A,B). Also, when the cell prepares for movement, there is a reorganization of the actin filaments, assessed by measuring F-actin using a FITC-phalloidin probe. CSE induced an increase in F-actin, while FO was able to restore basal levels of F-actin expression (Figure 6C). These findings highlight that FO could have a role in counteracting the effect of CSE on the migration of lung cancer cells. Furthermore, CSE increased the colony number, while pre-incubation with FO was able to counteract the effect of CSE on colony formation (Figure 6D).

## 3. Discussion

Epithelial-mesenchymal transition (EMT) has a crucial, relevant role in embryonic development, chronic inflammation, and cancer metastasis, and there is increasing evidence that considers it a central pathophysiological factor in COPD and lung cancer progression [33]. Patients with COPD have a higher risk of lung cancer. Smoking increases oxidative stress, inducing DNA damage and playing an important role in the pathogenesis of lung cancer [34]. Furthermore, mitochondrial dysfunction in patients with COPD is associated with excessive mitochondrial ROS levels, which contribute to enhanced inflammation and cell hyper-proliferation [35]. Inhalation therapy with LABAs, including FO, is indicated for the regular treatment of symptomatic patients with moderate-to-severe COPD, and FO reduces the mitochondrial oxidative stress induced by cigarette smoke exposure in non-tumorigenic bronchial epithelial cell lines [21]. A previous study [36] has demonstrated that patients with COPD and lung cancer who assume inhalation therapy with bronchodilators with/without corticosteroids have a significantly longer median overall survival. Based on this, in the present in-vitro study performed in an adenocarcinoma cells line (A549 cells) exposed to CSE, we have shown for the first time that FO is able to reduce intracellular and extracellular ROS and IL-8 release. Furthermore, it can counteract the effect of cigarette smoke on the EMT process and cell migration and proliferation, supporting the potential use of FO as an add-on treatment for lung cancer management. 

COPD and lung cancer are characterized by high oxidative stress [34] due to exposure to exogenous factors, such as cigarette smoke and environmental pollutants, but they are also generated endogenously by inflammatory cells. ROS plays a pro-tumorigenic effect and promotes cancer initiation, proliferation, and angiogenesis [37]. CSE increases intracellular ROS in normal human bronchial cell lines [20]. In particular, mitochondria are the major sources of oxidative stress in COPD [38]. We also evaluated the superoxide mitochondria in adenocarcinoma cell lines, and we observed that CSE increases mitochondrial superoxide production. Mitochondrial dysfunction is associated with the reduction of intracellular ATP [38], and we observed that FO counteracted the CSE effect on intracellular ATP production. ROS includes superoxide anions and hydrogen peroxide (H_2_O_2_). H_2_O_2_ has a longer half-life and can cross the biological membranes [39] by accumulating in the extracellular space and creating an environment that favors the formation of tumors [40]. CSE increases the extracellular ROS in adenocarcinoma cells, confirming the data obtained with flow cytometry. The quantification of H_2_O_2_, by using a specific electrochemical sensor only for H_2_O_2_, in cellular supernatants of A549 is a convenient, easy, and validated way to monitor the oxidative stress of the cell [27]. 

In our study on A549 cells, we showed that FO can reduce the intracellular and extracellular ROS, counteracting the pro-oxidant effect of CSE. In this way, FO could have a protective effect on downstream events related to oxidative stress. High levels of ROS activated pro-inflammatory signaling pathways and the release of multiple inflammatory mediators. Specifically, IL-8 plays a crucial role because it is closely related to oxidative stress and tumorigenic processes [15,16]. Tumor cells, through the expression of various chemokines and receptors, can promote their growth and survival in an autocrine and paracrine way. IL-8, in an autocrine way, promotes oncogenic signaling, angiogenesis, and invasive mechanisms in tumor cells. On the other hand, IL-8 affects the immune cells within the tumor microenvironment in a paracrine way [41]. Previous studies demonstrated that IL-8 is a good marker of neoplastic transformation because it is constitutively expressed at high levels in cancer airway epithelial cells compared to non-cancer airway epithelial cells [16]. FO was able to suppress the release of IL-8 in adenocarcinoma cells induced by TNF-α [42]. To our knowledge, there is no evidence relative to the effect of FO in cancer cells exposed to cigarette smoke. In the present in-vitro study, we assessed that CSE increased IL-8 release in adenocarcinoma cells, as well as observed for the first time that FO counteracts the effect of CSE in reducing IL-8 release. The interaction of FO with β2-adrenergic receptors causes the activation of the adenylate cyclase enzyme and increases cyclic adenosine monophosphate (cAMP) levels [19]. Forskolin is a compound capable of activating the adenylate cyclase enzyme and increasing cAMP levels. As such, it can be used as an internal control for the activity of the FO. This compound can exert both antioxidant and anti-inflammatory activities [43,44]. A549 cells treated with CSE and forskolin tended to release less IL-8 than CSE-stimulated cells, mimicking the action of FO.

The EMT process may be one of the connections between COPD and lung cancer, also affecting the progression, migration, and metastasis of cancer. During the EMT process, the epithelial cells undergo a structural change with a reduction of the expression of adhesion proteins, such as E-cadherin. Perpetuated exposure to cigarette smoke is associated with lung barrier damage, chronic inflammation that leads to abnormal tissue repair process, and transformation of epithelial cells in a more invasive mesenchymal phenotype [5]. We confirmed the reduction of the expression of E-cadherin in adenocarcinoma cells exposed to CSE [26], a protein involved in cell-cell adhesion and a typical epithelial marker used to study the EMT process. Herein, we demonstrated that FO could counteract the effect of CSE by increasing the expression of E-cadherin. The effect of FO was confirmed by forskolin, which has a similar capacity to increase the expression of E-cadherin after exposure to CSE, supporting the hypothesis that the activation of β2-adrenergic receptors could be a mechanism by which the effects of CSE on the EMT process could be counteracted. 

The EMT process in lung cancer is regulated by many transcription factors; for example, ZEB1, SNAIL1, and TWIST are considered the main regulators of EMT [45], and they are closely related to lung tumor progression [46]. CSE has a relevant role in inducing an increase in the expression of SNAIL1 in adenocarcinoma cells [26]. A previous study demonstrated that smokers and those with COPD expressed high levels of SNAIL1 [47], and the expression is related to the EMT process and airway obstruction [47]. Furthermore, the expression of E-cadherin is negatively regulated by SNAIL1 [31]. In this study, we confirmed that CSE up-regulated SNAIL1 expression in adenocarcinoma cells, and we observed for the first time that FO is able to reduce SNAIL1 expression and counteract the effect of CSE on the EMT process. Tumor development is also related to the ability of the cell to acquire migratory and invasive properties; in this context, EMT represents the first step of the switch from a non-motile epithelial to a motile mesenchymal state. CSE promotes cell migration and proliferation. This is confirmed by increasing actin reorganization and a scratch assay assessing an increase of cell migration, while a clonogenic assay assessed that CSE induced colony formation. In this context, we are demonstrating that FO in A549 cells counteracts the invasive, migratory, and proliferative events induced by CSE.

In conclusion, all data in the present study support the central role of CSE in inducing oxidative stress, inflammation, the EMT process, migration, and proliferation in lung adenocarcinoma cells and confirm the numerous data existing in the literature that correlate oxidative stress, the EMT process, and tumorigenesis in the lung [5,6,9,11,25,26,29]. These events could make COPD patients more susceptible to developing lung cancer. Long-acting β2-agonists are usually used in treating COPD patients, but its effects on the tumorigenic process are unknown. The novelty of this study is to show for the first time the effects of FO in relation to this process. In this in-vitro study that used a simple model of tumor cells from the adenocarcinoma cell line A549, we assessed for the first time that FO is able to counteract the effects of cigarette smoke related to oxidative stress, inflammation, the EMT process, migration, and proliferation, assuming a new potential role for COPD patients in protecting from lung cancer development. Among the limitations of this study is the use of a simple model of tumor cells, but this must be seen as a preliminary study to evaluate the effects of FO. These findings must be validated in the future by using a more complex model of epithelial cells, such as the Air-Liquid Interface (ALI). Furthermore, the results of the present study have potential translational outcomes that deserve validation in clinical practice by setting clinical studies to support the repositioning of FO as an add-on treatment for lung cancer management.

## 4. Materials and Methods

### 4.1. Cell Culture and Treatment

The A549 (CCL-185) (adenocarcinomic human alveolar basal epithelial cells) cell line was purchased from the Interlab Cell Line Collection (Genoa, Italy). The cells were cultured in humidified ambient air with 5% CO_2_ at 37 °C. A549 cells were maintained in RPMI-1640 medium supplemented with heat deactivated (56 °C, 30 min) 10% FBS, 1% streptomycin and penicillin, 1% non-essential amino acids, and 2 mM L-glutamine. Cells were grown to confluence and were stimulated with or without CSE 2.5% and Formoterol 10^−8^ M (F9552—Sigma-Aldrich, St. Louis, MO, USA) or Forskolin (FORSK) 20 μM (sc-3562 Santa Cruz Biotechnology, Dallas, TX, USA). FO and FORSK were added 30 min before CSE cell stimulation. During the treatment, the serum concentration in the medium was limited to 1%. The concentration of CSE and FO were chosen based on previous work [21,26] and preliminary experiments, Figure S1 of Supplementary Materials. The experiments were repeated at least three independent times, and each experiment was performed at least in duplicate.

### 4.2. Preparation of Cigarette Smoke Extracts (CSE)

A peristaltic pump, Watson-Marlow 323 E/D (Rotterdam, The Netherlands), was used to prepare cigarette smoke extract (CSE) as previously described [20]. Kentucky 3R4F research-reference cigarettes (The Tobacco Research Institute, University of Kentucky, Lexington, KY, USA) were used without the filter. Briefly, each cigarette was smoked for 5 min, and two cigarettes were used for 20 mL of phosphate-buffered saline (PBS) to generate a CSE-PBS solution. The CSE solution was filtered through a 0.22 μm pore filter to remove bacteria and large particles. The smoke solution was set up fresh for each independent experiment and was used at most within 30 min of its preparation. This solution, considered 100% CSE, was then diluted to obtain the desired concentration for each experiment. The concentration of CSE was verified spectrophotometrically, measuring the OD as previously described [48] at a wavelength of 320 nm. The pattern of absorbance among different batches showed very little difference, and the mean OD of the different batches was 1.4 ± 0.14.

### 4.3. Measure of Intracellular Reactive Oxygen Species (ROS)

All species of intracellular ROS were measured by flow cytometry, thanks to the conversion of the 6-carboxy-2′,7′-dichlorodihydrofluorescein diacetate (H2DCFDA, C-2938—Life Technologies, Carlsbad, CA, USA) nonfluorescent compound in DCF fluorescent compound [49]. A549 cells were seeded in a 12-well plate until 80–90% confluency; then, the A549 cells were treated with FO 10^−8^ M for 30 min. Next was added CSE 2.5% for 24 h. After the stimulation, the cells were harvested, washed with PBS, and labeled in the dark with 1 μM H2DCFDA for 30 min at room temperature (RT). At the end of the incubation, the cells were rinsed with PBS and analyzed by flow cytometry using CytoFLEX (BeckmanCoulter, Brea, CA, USA). The results are expressed as a percentage of positive cells.

### 4.4. Measure of Mitochondrial Superoxide

MitoSOX™ Red mitochondrial superoxide indicator (Molecular Probes Waltham, MA, USA) was used to evaluate the production of mitochondrial superoxide [21]. A549 cells were seeded in a 12-well plate until 80–90% confluency, then the A549 cells were treated with FO 10^−8^ M for 30 min, then was added CSE 2.5% for 24 h. Then, the cells were collected, rinsed with PBS, and labeled with a 3 μM MitoSOX Red probe for 15 min at 37 °C. At the end of the incubation, the cells were rinsed twice in PBS and then analyzed by flow cytometry using CytoFLEX (BeckmanCoulter). See the gating strategy in Figure S2 of Supplementary Materials. The results are expressed as a percentage of positive cells.

### 4.5. Measure of Intracellular ATP

Intracellular ATP was measured using the ATPlite Luminescence Assay System (PerkinElmer, Waltham, MA, USA) according to the manufacturer’s instructions. A549 cells were seeded in a white 96-well plate until 80–90% confluency; then, the A549 cells were treated as previously described. After 24 h of stimulation, a cell lysis solution was added, and after 5 min, the substrate solution was added. The luminescence was measured using GloMax^®^ Discover Microplate Reader (Promega Corporation, Madison, WI, USA). Results are shown as Relative Luminescence Units (RLU).

### 4.6. Measure of Extracellular ROS by Electrochemical Sensor 

Extracellular ROS was measured by the electrochemical quantification of hydrogen peroxide (H_2_O_2_). An electrochemical sensor was used based on reduced graphene oxide, rGO, and gold nanoparticles, AuNPs. The sensor was obtained by electrochemical co-deposition following the method detailed in our previous work [50]. In particular, on a flexible conductive substrate (polyethylene terephthalate covered with indium tin oxide), a thin layer of rGO and AuNPs was electrodeposited. This thin layer is the active material of the electrochemical sensor able to catalyze the electrochemical reduction of hydrogen peroxide. The hydrogen peroxide quantification was performed in a three-electrode cell consisting of a working electrode, the sensor, a counter, a platinum mesh, and an Ag/AgCl reference electrode. A 3D printed cell was used to ensure the analysis of a very small volume of sample, about 1 mL. The detection of hydrogen peroxide was carried out using amperometry as the electrochemical technique at the fixed potential of −0.8 V vs. Ag/AgCl. The current density was measured after the stabilization of the electrochemical signal. The stabilization point was selected by monitoring the slope of the chronoamperometric curves. The value was considered stable when a slope equal to or lower than 1 μA/s was reached. The stable value of current density was converted to hydrogen peroxide concentration using the calibration line of the sensor. Hydrogen peroxide was quantified in the medium of cellular growth (RPMI-1640) diluted in PBS (50% in volume). Prior to sample analysis, the sensor was calibrated using the PBS as a blank, where an increasing amount of hydrogen peroxide was progressively inserted. The linear range is 25–1000 μM with a limit of detection of 6.5 μM and a sensitivity of 0.0704 ± 0.0045 μA μM^−1^ cm^−2^. The analysis was performed at room temperature. Each test was carried out at least 3 times with 3 different electrodes.

### 4.7. Flow Cytometry

E-cadherin expression was evaluated by flow cytometry using a CytoFLEX (Beckman Coulter, Brea, CA, USA). A549 cells were treated with FO 10^−8^ M or FORSK 20 μM for 30 min. Next was added CSE 2.5% for 48 h, as indicated in previous work [26]. A549 cells were incubated with E-cadherin antibody (sc-21791 Santa Cruz Biotechnology, Dallas, TX, USA) followed by anti-mouse IgG FITC (Dako—Agilent Technologies, Santa Clara, CA, USA). See the gating strategy in Figure S2 of Supplementary Materials. Negative controls were set up using an isotype control antibody (BD PharMingen, Mountain View, CA, USA). Data are expressed as percentages of positive cells.

### 4.8. Determination of Actin Reorganization

A FITC–Phalloidin probe (200 ng/mL) (P5282 Sigma-Aldrich, St. Louis, MO, USA), which binds F-actin, was used for assessing via flow cytometry the actin reorganization as previously described [51]. A549 cells were treated with FO 10^−8^ M for 30 min. Then CSE 2.5% was added for 48 h, and the cells were fixed (4% paraformaldehyde in PBS), permeabilized (0.1% Triton X-100 in PBS), and incubated with the fluorescent dye in the dark at RT for 30 min. CytoFLEX (Beckman Coulter, Brea, CA, USA) was used for the flow cytometry analyses, and data are expressed as percentages of positive cells. See the gating strategy in Figure S2 of Supplementary Materials.

### 4.9. Measurement of IL-8

The concentrations of IL-8 were measured with enzyme-linked immunosorbent assay (ELISA) (Duo Set R&D Systems, Minneapolis, MN, USA) according to the manufacturer’s instructions. The data are expressed as pg/mL.

### 4.10. Cell Migration Scratch Assay

The scratch assay evaluates cell migration following the creation of a cell-free zone (wound) in the cell monolayer. A549 cells were cultured in a 6-well plate to confluence. Three circular wounds were made in each well using a 200-μL pipette tip. After rinsing with PBS to remove debris, cells were allowed to recover for one hour and then pre-incubated with FO 10^−8^ M for 30 min and exposed to CSE 2.5%, with or without FO for 24 h. An inverted phase-contrast light microscope connected to a digital camera was used to collect digital images at times 0, 24, and 48 h after scratching. The Image J program was used to measure the surface of the wound area to assess the remaining wound size and wound closure rates. The results were expressed as a percentage of area reduction at time point 24 h (T24-T0) and 48 h (T48-T0) compared to time point 0 h (T0h), with each specific treatment having its own time zero as reference.

### 4.11. Clonogenic Assay

After CSE 2.5% treatment (24 h) with or without FO 10^−8^ M (FO was added 30 min before CSE cell stimulation), the A549 cells were collected and cultured in a 6-well plate at a clonogenic density of 50 cells/cm^2^ to form colonies in 1–3 weeks. During the colony formation period, cells were cultured in a fresh medium at 37 °C in an atmosphere containing 5% CO_2_. After the time necessary for the formation of colonies with at least 50 cells, the cells were fixed in 100% methanol and labeled with 0.5% crystal violet in 20% methanol. Thereafter, the plates were air-dried, and the colonies were photographed using a digital camera and counted by using image master 2D count PHICS (count and Plot Histograms of Colony Size) software, a macro written for Image J (https://imagej.net, accessed on 8 June 2023) [52].

### 4.12. Real-Time PCR

TRIzol Reagent (Life Technologies) was used following the manufacturer’s instruction for total RNA extraction from A549 cells stimulated for 24 h as previously described. Briefly, 1 μg of RNA was reverse transcribed to cDNA using an iScript cDNA Synthesis kit (Biorad). E-cadherin gene (CDH1), SNAIL1 gene expression was assessed by qRT-PCR conducted at Step One Plus Real-time PCR System (Applied Biosystems, Foster City, CA, USA) using specific FAM-labeled probe and primers (prevalidated TaqMan Gene expression assay for CDH1, Hs001023895_m1; for SNAIL1, Hs00195591_ml; Applied Biosystems) as previously described [21,53]. Gene expression was normalized to GAPDH (prevalidated TaqMan Gene expression assay for GAPDH, Hs03929097_g1) as an endogenous control gene. The relative quantification of mRNA was obtained with the comparative Ct method (2^−ΔΔCt^) and was plotted as a fold-change compared to untreated cells that were chosen as the reference sample.

### 4.13. Statistics

Experiments were repeated at least three times. Data are expressed as mean ± SD and analyzed by ANOVA followed by the Bonferroni post hoc test by using GraphPad Prism 9. A *p*-value < 0.05 was considered statistically significant.

## Data Availability

Data is contained within the article.

## Supplementary Materials

The supporting information can be downloaded at: https://www.mdpi.com/article/10.3390/ijms242216088/s1.

## Abbreviations

CS	Cigarette smoke
CSE	Cigarette smoke extract
ROS	Reactive oxygen species
COPD	Chronic Obstructive Pulmonary Disease
GOLD	Global Initiative for Chronic Obstructive Lung Disease
FO	Formoterol
FORSK	Forskolin
EMT	Epithelial-to-mesenchymal transition
CDH1	E-cadherin gene
SCLS	Small-cell lung cancer
NSCLS	Non-small-cell lung cancer
LABAs	Long-acting β2-agonists
