# Supplementary material for: Formoterol Exerts Anti-Cancer Effects Modulating Oxidative Stress and Epithelial-Mesenchymal Transition Processes in Cigarette Smoke Extract Exposed Lung Adenocarcinoma Cells
Source: Int J Mol Sci. 2023 Nov 8;24(22):16088. doi: 10.3390/ijms242216088 (PMC10671675; doi:10.3390/ijms242216088)
Supplement: Supplementary file 1 [file ijms-24-16088-s001.zip › ijms-2470483-supplementary.pdf]

Supplementary Materials

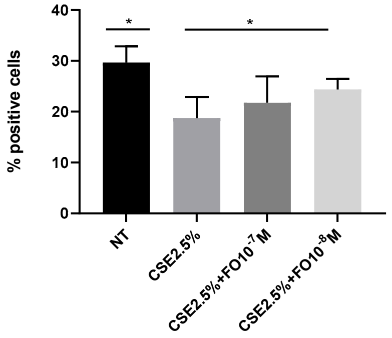

Figure S1. E-cadherin protein expression in A549 cells, dose response experiment. A549 cells were exposed to CSE 2.5% with or without FO 10^{-7} M or FO 10^{-8} M for 48 h, FO were added 30 min before CSE cell stimulation. E-cadherin protein expression was evaluated by flow cytometry. E-cadherin protein expression in A549 cells was showed as % positive cells and expressed as mean ± SD (*N* = 3). Representative histograms relative E-cadherin protein expression (**B**–**D**) were shown. **p* < 0.05 ANOVA with Bonferroni correction.

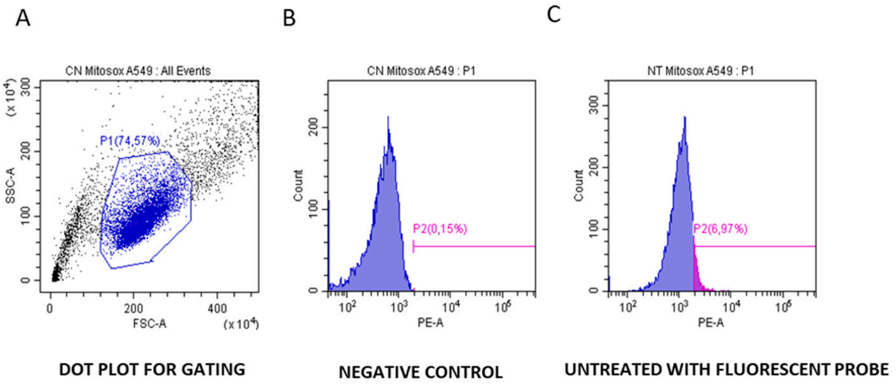

Figure S2. Gating strategy. (**A**) Representative dot plot relatively morphologic parameters FSC (forward scatter) and SSC (side scatter), with gate region P1; (**B**) Representative histogram of negative control; (**C**) Representative histogram with fluorescent probe.

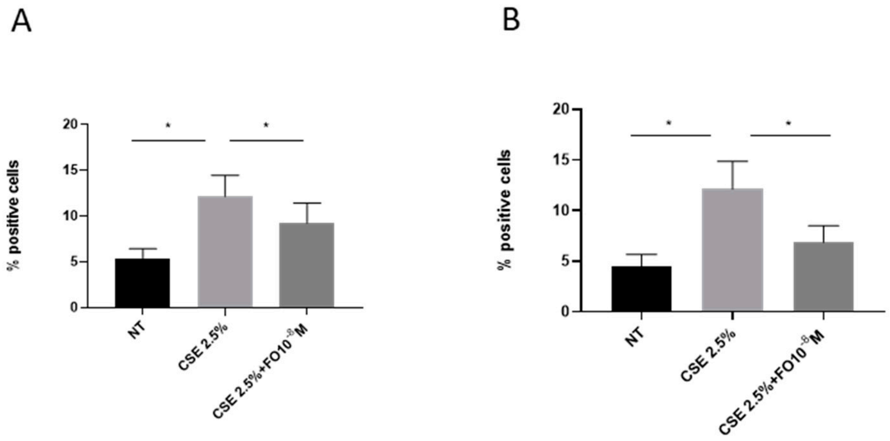

Figure S3. ROS and mitochondrial superoxide production in COLO699 N cells. COLO699 N cells were exposed to CSE 2.5% with or without FO 10^{-8} M for 24h, FO was added 30 min before CSE cell stimulation. ROS, and mitochondrial superoxide were assessed by flow cytometry. ROS (**A**) and mitochondrial superoxide (**B**) levels in COLO699 N cells were showed as % positive cells and expressed as mean ± SD (*N* = 5). **p* < 0.05 ANOVA with Bonferroni correction.

Supplementary Materials and Methods

Cell Culture COLO699 N

COLO699 N (adenocarcinoma cells derived from pleural fluid), cell line was purchased from Interlab Cell Line Collection (Genoa, Italy). The cells were cultured in a humidified ambient and air with 5% CO_2 at 37 °C. COLO699 N were maintained in RPMI-1640 medium supplemented with heat deactivated (56 °C, 30 min) 10% FBS, 1% streptomycin and penicillin, 1% non-essential amino acids and 2 mM L-glutamine. Cells were grown to confluence and were stimulated with or without CSE 2.5% and Formoterol 10^{-8} M (F9552- Sigma-Aldrich, St. Louis, Missouri, USA). Formoterol (FO) was added 30 min before CSE cells stimulation. During the treatment, serum concentration in the medium was limited to 1%. The experiments were repeated at least three independent times and each experiment was performed at least in duplicate.
